# Editorial: Highlights in personality and social psychology: life satisfaction

**DOI:** 10.3389/fpsyg.2023.1289803

**Published:** 2023-10-09

**Authors:** Pninit Russo-Netzer, Dan-Cristian Dabija, Bianca P. Acevedo

**Affiliations:** ^1^School of Advanced Studies, Achva Academic College, Shikmim, Israel; ^2^Department of Marketing, Faculty of Economics and Business Administration, Babes-Bolyai University Cluj-Napoca, Cluj-Napoca, Romania; ^3^Department of Psychological and Brain Sciences, University of California, Santa Barbara, Santa Barbara, CA, United States

**Keywords:** life satisfaction, well-being, cross-cultural, development, relationships, satisfaction with life (SWL)

Life satisfaction (LS) is a multifaceted concept that encompasses various domains and may be influenced by a wide range of factors, including personality traits, relationship dynamics, and socio-environmental circumstances. Essentially, it reflects the extent to which individuals assess their overall contentment with their lives (Diener, 1984), often considered as a general, overarching well-being indicator (e.g., Lounsbury et al., 2005). In recent decades, interest in LS has grown substantially (Emerson et al., 2017), yielding insights regarding its various positive outcomes, such as increased income and improved health (Diener et al., 2018; Lombardo et al., 2018). However, more knowledge is needed to delve into the underlying psychological processes that contribute to higher life satisfaction (Diener et al., 2017), especially during and in post-crisis situations (e.g., the COVID-19 pandemic outbreak, the armed conflict in Eastern Europe, the energy and resources crises in recent months, and more) which impact us as individuals and societies. Furthermore, research is needed to properly assess the impact of various personal and professional levers on life satisfaction, such as well-being, task performance, contextual performance, work engagement, teleworking, counterproductive work-behavior, professional isolation or turnover intention.

## New perspectives and directions in the study of life satisfaction

In this Research Topic, articles shed light on the multifaceted nature of life satisfaction and provide valuable insights into its various factors, determinants and outcomes. By exploring the potential roles of personality, relationships, and external circumstances, the studies contribute to a more holistic understanding of well-being. The first paper, by Malvaso and Kang, presents a comprehensive examination of the connection between personality traits, different domains of life satisfaction, and the overarching sense of well-being. The research underscores the importance of various personality traits in shaping an individual's perception of life satisfaction. By integrating these factors, the study offers a nuanced perspective on how personality traits can influence different areas of life satisfaction, ultimately contributing to an individual's overall well-being. This integrated approach paves the way for tailored interventions aimed at enhancing life satisfaction based on personality profiles.

Interpersonal relationships also play an important role in shaping an individual's life satisfaction. The second paper, by Preetz, focuses on a specific aspect of relationships—the dissolution of non-cohabiting relationships—and its impact on life satisfaction and mental health. The findings shed light on the potential challenges and the emotional toll associated with the end of such relationships. By highlighting the connection between relationship changes and psychological well-being, this research underscores the need for effective support systems and coping strategies during periods of relationship transition, aiming to mitigate potential negative impacts on life satisfaction.

Other potential factors may involve the interplay between internal and external conditions, such as gender disparities in life satisfaction. The third paper, by Eriksson and Strimling highlights how gender differences in competitiveness and fear of failure contribute to variations in life satisfaction, particularly among girls in gender equal countries. The study draws attention to the role of societal norms and gender-specific pressures in shaping life satisfaction outcomes, offering insights for interventions aimed at addressing these disparities.

Body image also plays a pivotal role in shaping self-perception and overall life satisfaction. This issue is explored in depth in the fourth paper, by Wagner and Singh. This paper delves into consumers' body image expressions and their psychological implications. By exploring the contrast between aspiring to a “Snow White” or an “Evil Queen” archetype, the research unveils the intricate relationship between consumer behavior, body image ideals, and their impact on life satisfaction.

When exploring new frontiers in the study of life satisfaction, several papers in this Research Topic underscore unique environmental circumstances and populations. The COVID-19 pandemic brought about unprecedented global changes, including lockdown measures that affected various aspects of daily life. The fifth paper, by Chen et al. delves into the impact of relative deprivation—the perception of having less than others—on life satisfaction during the COVID-19 lockdown. Through serial mediation analyses, the study reveals the complex pathways through which relative deprivation can influence life satisfaction. These findings contribute to our understanding of how societal and economic disparities can exacerbate the impact of external stressors on individual well-being. The research calls for targeted interventions to address relative deprivation, especially during times of crisis, to promote and sustain life satisfaction.

The sixth paper focuses on the population of Psychiatric inpatients. The paper, by Chi et al. explores psychiatric inpatients and their experience of life satisfaction. By examining the relationships between positive schemas—cognitive frameworks that shape our perceptions—and life satisfaction, the research provides valuable insights into the inner workings of individuals grappling with mental health challenges. The findings emphasize the role of positive cognitive patterns in fostering life satisfaction even in the context of psychiatric care, highlighting the potential of cognitive interventions to enhance well-being among this population.

The seventh paper is related to the importance of a stable and suitable living environment to life satisfaction. The paper, by Hu et al. delves into the intricate interplay between housing difficulties, health status, and overall life satisfaction. The study underscores the need for adequate housing conditions, as housing difficulties are shown to have cascading effects on both physical health and psychological well-being, thereby influencing life satisfaction outcomes.

Finally, the eight paper, by Russo-Netzer and Icekson explores a rather underexplored pathway to life satisfaction—synchronicity, the meaningful coincidence of events. This study introduces the Synchronicity Awareness and Meaning-Detecting Scale, uncovering a novel link between synchronicity awareness and life satisfaction. By validating this scale, the research pioneers a deeper exploration of the profound connections between meaningful experiences and overall life satisfaction, expanding our understanding of how the recognition of synchronicity can contribute to a more fulfilling life.

Overall, the articles in this Research Topic collectively contribute to broadening and deepening our understanding of the multifaceted nature of life satisfaction. They underscore the diverse array of factors that influence our well-being, specifically life satisfaction. As we navigate the complexities of life, these findings underscore the importance of tailored interventions, social support networks, and the environment to enhance life satisfaction across diverse populations. As researchers continue to explore these facets, a more comprehensive picture of what contributes to a fulfilling life emerges, offering guidance for both individuals and policymakers striving to promote well-being and life satisfaction.

## Author contributions

PR-N: Conceptualization, Writing—original draft, Writing—review and editing. D-CD: Writing—review and editing. BA: Writing—review and editing.
